# Correction to “Different SARS‐CoV‐2 Subvariants and Coagulation Markers Trend: A Retrospective Multicentric Study From Iranian Network for Research in Viral Diseases (INRVD)”

**DOI:** 10.1002/hsr2.72568

**Published:** 2026-05-25

Razavi A, Koltapeh MP, Nazari T, Morvaridi A, Banivaheb B, Saeb S, et al. Different SARS‐CoV‐2 Subvariants and Coagulation Markers Trend: A Retrospective Multicentric Study From Iranian Network for Research in Viral Diseases (INRVD). *Health Science Reports*. 2026;9(4):e72390.

The affiliation of the corresponding author, Seyed Mohammad Jazayeri, was incorrectly linked to affiliation 2. The correct affiliation is affiliation 1: “Research Center for Clinical Virology, Tehran University of Medical Sciences, Tehran, Iran.”

The online version of this article has been corrected accordingly.

We apologize for this error.

